# Bridging the Oxygenation Gap: A Dual-Modality Approach in Severe Acute Respiratory Distress Syndrome

**DOI:** 10.7759/cureus.104230

**Published:** 2026-02-25

**Authors:** Nafeesathu Misiriyyah, Humaid Sadiq, Tarab Iqbal, Lajeesh Vettikkat

**Affiliations:** 1 Emergency Medicine, Tawam Hospital, Al Ain, ARE

**Keywords:** acute respiratory distress syndrome [ards], apneic oxygenation, high-flow nasal canula, noninvasive ventilation for preoxygenation, peri intubation oxygenation

## Abstract

Patients suffering from severe acute respiratory distress syndrome (ARDS) are at an increased risk for peri-intubation complications due to profound hypoxemia, poor lung compliance, and intrapulmonary shunting. Standard preoxygenation methods often leave patients vulnerable to rapid desaturation during apnea. A dual-modality preoxygenation strategy combining noninvasive ventilation (NIV) and high-flow nasal cannula (HFNC) has emerged as a physiologically sound approach to tackle this challenge.

We report a case of a 46-year-old gentleman with no known comorbidities who presented with severe dyspnea and respiratory failure secondary to necrotizing pneumonia. Despite maximal oxygenation via a non-rebreather mask, peripheral capillary oxygen saturation (SpO₂) remained below 90%, necessitating prompt endotracheal intubation. Preoxygenation was achieved with NIV using a bilevel positive airway pressure (BiPAP) device, followed by HFNC at 60 L/min during induction. The patient maintained a SpO₂ > 94% throughout the peri-intubation period without any complications.

This case highlights that combining NIV and HFNC can effectively bridge the oxygenation gap in severe ARDS, minimizing peri intubation hypoxemia, the severe oxygen desaturation during intubation, and enhancing patient safety during emergent airway management. This approach could be considered in profoundly hypoxemic patients requiring intubation in an emergency setting.

## Introduction

Endotracheal intubation in patients with severe ARDS is a race against rapidly worsening hypoxemia. The severe inflammatory process in ARDS causes endothelial and cellular damage, causing alveolar collapse. Profound oxygen deficits, extensive intrapulmonary shunting, and severely reduced lung reserves leave little margin for error, with even brief periods of apnea capable of precipitating rapid desaturation and catastrophic hypoxic injury. This mandates the use of positive end-expiratory pressure (PEEP) in addition to oxygen supplementation while oxygenating these patients to improve alveolar recruitment and counteract shunt physiology. Despite advances in airway techniques and monitoring, peri-intubation hypoxemia remains a persistent and potentially fatal complication in this population [1,2].

The cornerstone of safe airway management in severe ARDS is effective oxygenation [1,2]. During rapid sequence intubation (RSI), standard face masks and non-rebreather masks (NRBM) need to be removed prior to the introduction of a laryngoscope, which leaves patients without oxygen during apnea and increases the risk of rapid desaturation. A low-flow nasal cannula can remain in place, but its limited flow provides minimal extension of the period of safe apnea [3]. This gap in oxygenation significantly increases the risk of peri-intubation hypoxemia and its potentially catastrophic consequences [1,2].

Noninvasive ventilation (NIV) and high-flow nasal cannula (HFNC) have emerged as valuable adjuncts for preoxygenation, each addressing distinct aspects of the underlying physiology. NIV improves oxygenation by increasing PEEP and promoting alveolar recruitment, thereby combating ventilation-perfusion mismatch [1,2]. HFNC delivers high-flow, heated, and humidified oxygen, facilitating dead-space washout, providing continuous apneic oxygenation nasally during RSI, and generating a modest PEEP [3]. Clinical studies have demonstrated that HFNC can prolong safe apnea period and reduce peri-intubation desaturation in critically ill patients [4,5]. Combining these modalities may offer synergistic benefits, optimizing oxygenation before laryngoscopy and potentially reducing the risk of peri-intubation hypoxemia [6,7].

This case report describes the successful implementation of a novel approach combining NIV and HFNC to bridge this critical oxygenation gap during rapid sequence intubation in a patient with severe ARDS. The complementary effects of these modalities maintain oxygenation during apnea, reduce peri-intubation hypoxemia, and provide a practical, physiologically grounded method to enhance airway safety in patients with severe hypoxemic respiratory failure [6,7].

## Case presentation

A 46-year-old gentleman with no known comorbidities presented to the emergency department (ED) with worsening shortness of breath and cough for one week, associated with nausea and epigastric pain. No recent illnesses were reported. Travel history was positive for a visit to his home country two weeks prior. He was a non-smoker with a normal body mass index. On physical examination, the patient was severely tachypneic with a respiratory rate of 35 breaths/min and an SpO_2_ of 80% on room air. Chest auscultation revealed reduced air entry over the right lower lobe and scattered coarse crackles bilaterally. There was mild tenderness in the right upper quadrant without guarding. 

An arterial blood gas was obtained after initiating oxygen supplementation via a non-rebreather mask (NRBM) ramped up to a maximum flow of more than 15L/min. It showed respiratory acidosis, hypoxemia, and hypercarbia, consistent with type 2 respiratory failure. The PaO_2_/FiO_2_ (PF) ratio was markedly reduced to 68, considering an FiO2 of 95%, indicating severe ARDS. Serum lactate and inflammatory markers, including C-reactive protein (CRP), were elevated. All biochemical test results obtained are summarized in Table 1.

**Table 1:** Relevant laboratory studies obtained on presentation. pH: potential of hydrogen, paCO2: partial pressure of carbon dioxide, paO2: partial pressure of oxygen, WBC: white blood cell.

Laboratory test	Laboratory value	Normal reference range
Arterial blood gas		
pH	7.14	7.35-7.45
paCO_2_	72.3 mmHg	35-45 mmHg
paO_2_	65 mmHg	80-100 mmHg
Bicarbonate	22 mmol/L	22-26 mmol/L
Complete Blood Count		
WBC count	28.8 x 10⁹/L	4.0-11.0 x 10⁹/L
Neutrophils	13 x 10⁹/L	2.0-7.5 × 10⁹/L
Platelet count	452 x 10⁹/L	150-400 × 10⁹/L
General Biochemistry		
Sodium serum	125 mmol/L	136-145 mmol/L
Potassium serum	3.8 mmol/L	3.3-4.8 mmol/L
Lactic acid serum	3.1 mmol/L	0.5-2.2 mmol/L
Creatinine serum	42 µmol/L	62-106 µmol/L
C-reactive protein	156 mg/L	0-5 mg/L
Procalcitonin	29.87 ng/mL	0-0.07 ng/mL
Lipase serum	12 IU/L	13-60 IU/L

**Figure 1:**
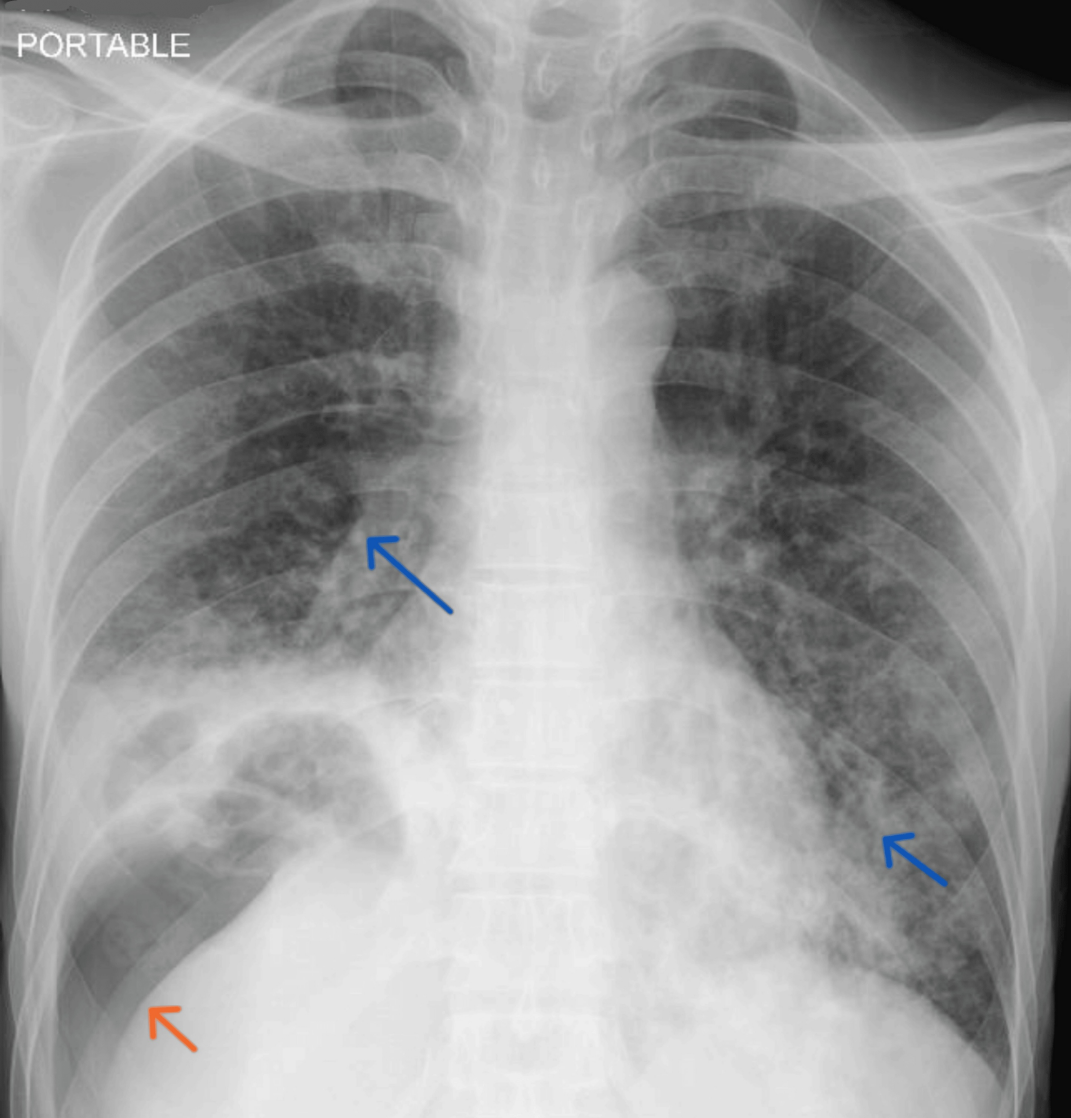
A portable bedside AP Chest X-ray demonstrates a dense consolidation with loculated air within the lower lobe of the right lung (orange arrow) and bilateral patchy infiltrates (blue arrow). AP: anteroposterior.

A bedside chest X-ray demonstrated bilateral pneumonic changes with loculated air in the right lower lobe (Figure 1).

**Figure 2:**
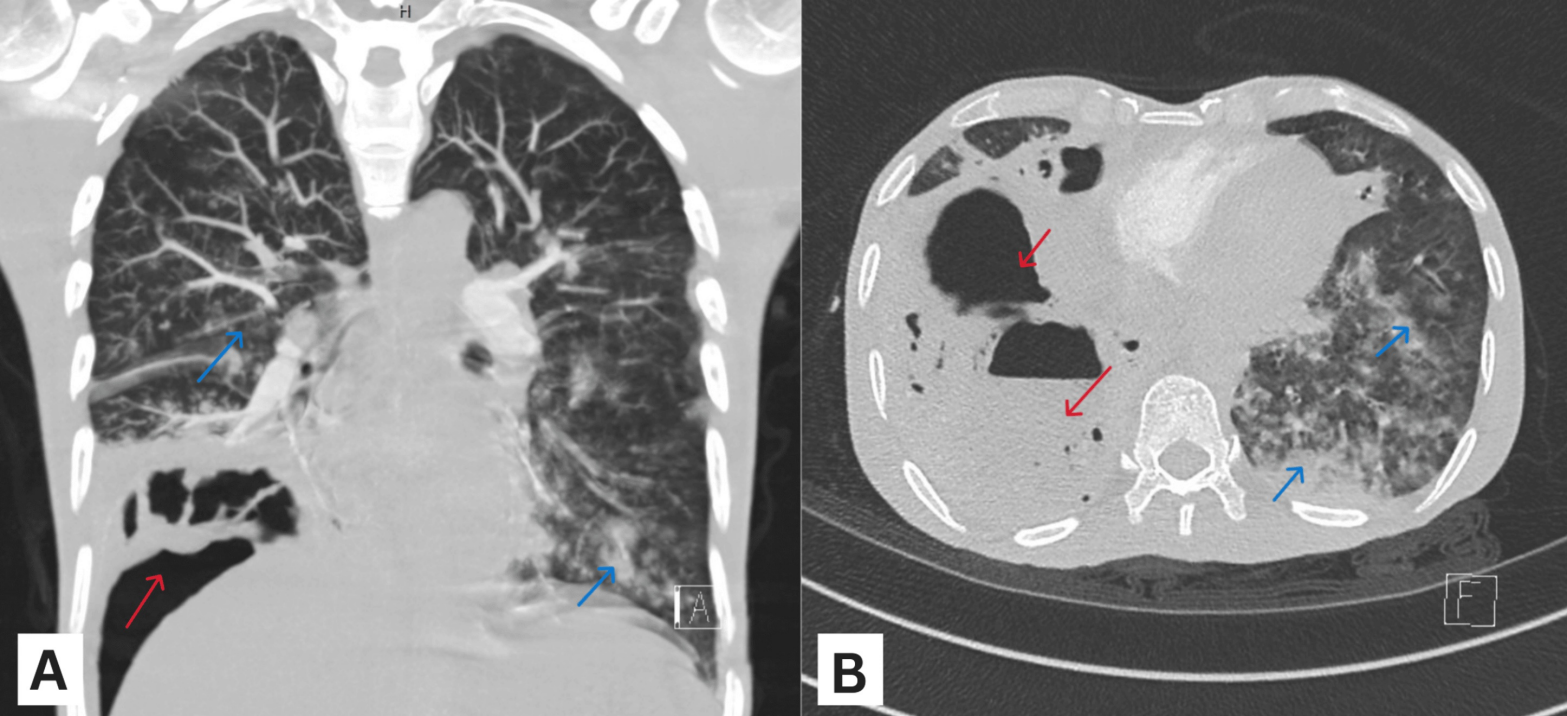
CT images showing the coronal view (A) and axial view (B) of a loculated cavitary consolidation with air fluid level in the lower lobe of the right lung (red arrows). Bilateral patchy infiltrates can also be seen (blue arrows). CT: computed tomography.

To rule out pneumoperitoneum secondary to hollow viscus perforation, a computed tomography (CT) scan of the chest and abdomen was performed, which revealed the presence of an aggressive necrotizing pneumonia with cavitation of the right lower lung (Figure 2). Imaging also confirmed diffuse bilateral infiltrates, consistent with the diagnosis of ARDS with a focal pathology. No evidence of bowel perforation was noted.

The patient was persistently desaturating to 88% despite receiving 15 L/min of oxygen via an NRBM. The decision for endotracheal intubation was taken in view of severe ARDS and the risk of an impending respiratory arrest, considering the patient was already in type 2 respiratory failure. However, the critical nature of the illness, combined with the patient's clinical condition, posed a significant risk for peri-intubation desaturation and subsequent hypoxia.

Broad-spectrum antibiotic coverage with a single dose of 4.5 g of intravenous (IV) piperacillin-tazobactam was given. A dual modality approach to preoxygenation and apneic oxygenation was utilized to manage this patient. NIV with a BiPAP device was applied for five minutes with a PEEP of 8 cmH2O. Though an end-tidal oxygen (EtO_2_) was not obtained, an SpO_2_ of 90% was achieved. Five minutes prior to induction, an HFNC delivering a continuous flow of 60 L/min of heated, humidified oxygen was initiated for apneic oxygenation. The BiPAP mask was removed after administration of 20 mg of IV etomidate and 60 mg of IV succinylcholine. The HFNC continued to provide apneic oxygenation while an endotracheal tube of 7.5 mm diameter was inserted by a second-year emergency medicine resident using direct laryngoscopy under direct vision in one attempt. The patient's SpO_2_ remained above 94% throughout an apnea period of 75 seconds, and no other complications occurred. Vital signs, including mean arterial pressure (MAP) and heart rate, were maintained within normal limits in the peri-intubation period without any vasopressor support. IV fentanyl and midazolam infusions were used for post intubation sedation, and the patient was transferred to the intensive care unit (ICU) for further management.

## Discussion

Severe ARDS presents a critical oxygenation challenge in the emergency department. Poor lung compliance and intrapulmonary shunting shorten safe apnea time [1,2]. Bridging this oxygenation gap is essential to prevent rapid peri-intubation desaturation. Preoxygenation strategies, particularly dual-modality approaches combining NIV with HFNC, aim to extend safe apnea time and improve patient safety [1,2,6,7].

NIV, particularly using a BiPAP device, supports oxygenation by recruiting collapsed alveoli, reducing ventilation-perfusion mismatch, and improving functional residual capacity (FRC) [1,2]. Studies have shown that NIV-assisted preoxygenation can significantly improve oxygen saturation before rapid sequence intubation in hypoxemic patients, outperforming standard bag-valve mask oxygenation [1,2]. However, once the NIV mask is removed for laryngoscopy, patients are at risk of rapid desaturation due to the depleted lung reserve volumes and interruption of oxygen delivery. This limitation is overcome by delivering a continuous high-flow supply of humidified oxygen using HFNC, even during apnea [3]. Apneic oxygenation with an HFNC maintains continuous oxygen flow and facilitates alveolar dead-space washout, while the modest PEEP it generates helps augment alveolar recruitment and improve FRC [3]. Recent studies have demonstrated that HFNC can prolong safe apnea time and reduce the incidence of peri-intubation desaturation in critically ill patients [4,5]. By addressing the oxygenation gap left when NIV is removed for laryngoscopy, HFNC serves as a complementary modality that enhances patient safety.

The combined use of NIV and HFNC offers the synergistic advantages of alveolar recruitment from NIV and continuous oxygen delivery from HFNC, optimizing oxygenation prior to laryngoscopy and maintaining it throughout the apneic period. In our case, this dual-modality strategy resulted in stable peri-intubation oxygen saturation without complications. Evidence from the literature supports this approach, with Jaber et al. demonstrating a significant reduction in severe desaturation during intubation of hypoxemic patients when NIV and HFNC were used together, compared to either modality alone [6]. Similarly, Frat et al. reported that combining these modalities may provide particular advantages in patients with ARDS and other forms of refractory hypoxemia [7].

The combination of NIV and HFNC offers a physiologically sound and clinically effective strategy for preoxygenation and apneic oxygenation in patients with severe ARDS. This case adds to growing evidence that a dual-modality approach can minimise peri-intubation hypoxemia and improve patient safety during emergency airway management. Despite supportive evidence, standardized guidelines for flow rates, timing, and transition between NIV and HFNC have not yet been established. As a single patient experience, the findings cannot be generalized or used to establish causation. In addition, the absence of a standardized institutional protocol for dual-modality preoxygenation may affect reproducibility across different clinical settings. Additionally, clear recommendations regarding optimal settings, timing, and duration of each modality remain limited, which may result in variability in clinical application.

## Conclusions

As the number of studies based on this dual-modality approach is limited, large randomized and blinded clinical trials are needed to define optimal protocols and assess potential complications. Additionally, the successful implementation of this practice in emergency and critical care settings depends on the clinician's familiarity with the technique and the availability of appropriate equipment. Addressing these factors will be essential to ensure safe, effective, and reproducible implementation across diverse clinical environments.

## References

[1] Baillard C, Fosse JP, Sebbane M (2006). Noninvasive ventilation improves preoxygenation before intubation of hypoxic patients. Am J Respir Crit Care Med.

[2] Mort TC (2005). Preoxygenation in critically ill patients requiring emergency tracheal intubation. Crit Care Med.

[3] Parke RL, McGuinness SP (2013). Pressures delivered by nasal high flow oxygen during all phases of the respiratory cycle. Respir Care.

[4] Miguel-Montanes R, Hajage D, Messika J (2015). Use of high-flow nasal cannula oxygen therapy to prevent desaturation during tracheal intubation of intensive care patients with mild-to-moderate hypoxemia. Crit Care Med.

[5] Vourc'h M, Asfar P, Volteau C (2015). High-flow nasal cannula oxygen during endotracheal intubation in hypoxemic patients: a randomized controlled clinical trial. Intensive Care Med.

[6] Jaber S, Monnin M, Girard M (2016). Apnoeic oxygenation via high-flow nasal cannula oxygen combined with non-invasive ventilation preoxygenation for intubation in hypoxaemic patients in the intensive care unit: the single-centre, blinded, randomised controlled OPTINIV trial. Intensive Care Med.

[7] Frat JP, Ricard JD, Quenot JP (2019). Noninvasive ventilation versus high-flow nasal cannula oxygen therapy with apnoeic oxygenation for preoxygenation before intubation of patients with acute hypoxaemic respiratory failure: a randomised, multicentre, open-label trial. Lancet Respir Med.

